# Cervical Mullerian Adenosarcoma with heterologous sarcomatous overgrowth: a fourth case and review of literature

**DOI:** 10.1186/1471-2407-11-236

**Published:** 2011-06-11

**Authors:** Tito Silvio Patrelli, Salvatore Gizzo, Stefania Di Gangi, Giorgia Guidi, Mario Rondinelli, Giovanni Battista Nardelli

**Affiliations:** 1 Department of Gynecological and Human Reproduction Sciences; University of Padua; via Giustiniani 3, 35128 Padua, Italy; 2 Department of Obstetrics, Gynecological and Perinatology Sciences; University of Parma, viale Gramsci 14, 43100 Parma, Italy

## Abstract

**Background:**

Uterine sarcomas are relatively rare tumors that account for approximately 1-3% of female genital tract malignancies and between 4-9% of uterine cancers. Less than 8% of all cases are Mullerian adenosarcoma, a distinctive uterine neoplasm characterized by a benign, but occasionally atypical, epithelial and a malignant, usually low-grade, stromal component, both of which should be integral and neoplastic constituents of the tumor. Mullerian adenosarcoma with sarcomatous overgrowth (MASO) is a very aggressive variant, associated with post-operative recurrence, metastases, even when diagnosed in early stage.

**Case Presentation:**

We present a fourth MASO case derived from uterine cervix in a 72-year-old woman with metrorrhagia and a polypoid mass protruding through the cervical ostium. Total abdominal hysterectomy, bilateral salpingo-oophorectomy, systematic pelvic lymph node dissection, omental biopsy and appendectomy were performed. Surgery treatment was associated with adjuvant whole-pelvis radiation (45 Gy) and adjuvant chemotherapy (cisplatin/ifosfamide). After nine months of follow up, the patient was free of tumor.

**Conclusions:**

The rarity of MASO of the cervix involves a management difficult. Most authors recommend total abdominal hysterectomy, usually accompanied by bilateral salpingo-oophorectomy. There is no common agreement on staging by lymphadenectomy during primary surgery and adjuvant chemo-radio therapy.

## Background

Uterine sarcomas are relatively rare tumors that account for approximately 1-3% of female genital tract malignancies and between 4-9% of uterine cancers[1-4]. Recently a new FIGO classification and staging system [5] has been specifically designed for uterine sarcomas to reflect their different biologic behavior. Three new classifications have been developed: staging for leiomyosarcomas and endometrial stromal sarcomas, staging for adenosarcomas, staging for carcinosarcomas.

Particular interest in this treatment is covered by Mullerian adenosarcoma, whose term was introduced by Clement and Scully [6] in 1974 for a distinctive uterine neoplasm characterized by a benign, but occasionally atypical, epithelial and a malignant, albeit usually low-grade, stromal component, both of which should be an integral and neoplastic constituent of the tumor. This tumor represents less than 8% of cases of uterine sarcomas[7-9]. Even rarer is the variant of Mullerian adenosarcoma with sarcomatous overgrowth (MASO). It is very aggressive and associated with post-operative recurrence, metastases, even when diagnosed and treated in early stages[7,10,11]. The stromal component may include only elements indigenous to uterus (homologous) or show differentiation toward elements not normally found in the uterus (heterologous), such as cartilage, osteoid and striated muscle[12,13]. Among these rhabdomyosarcoma is the most frequent. Adenosarcoma arise most commonly from the endometrium, sometimes including the lower uterine segment, but some cases are situated in the endocervix and rare examples arise within the myometrium from adenomyosis. More rarely, adenosarcoma occurs in the vagina, in the ovary, in the fallopian tube, arising from peritoneal surface, or outside the female genital tract, for example in the gut[14].

MASO of the uterine cervix is extremely rare. In this report we present a MASO case, derived from uterine cervix of a 72-year-old woman. To our knowledge it is the fourth case reported in the English literature for its location and the second MASO case of the cervix with heterologous elements.

## Case presentation

A 72-year-old Caucasian woman, Para 2012, in menopause from the age of 51 years, who had never taken hormone replacement therapy (HRT), presented with metrorrhagia from about 20 days. In physiological anamnesis the patient reported urinary incontinence and nocturia, she denied taking oral contraceptive in fertile-age. Pelvic examination revealed an elongated fleshy polyp, protruding through the cervical ostium, probably from endocervix. At the transvaginal ultrasonography revealed a polypoid mass subverting completely the uterine echostructure, with a thin residual posterior margin of intact myometrium. Intense neovascularization. The polypoid mass was removed for biopsy. Macroscopically, the lesion was measured as 3 × 1, 5 × 1, 5 cm. Microscopically, it was composed of a mucus-blood material encompassing lymphocytes, histiocytes and atypical elements of neoplastic nature. There were markedly anaplastic focal areas composed of pleomorphic spindle cell proliferation. About the immunophenotype, vimentin was positive and desmin was positive in focal areas, but keratin (MNF116) was negative. These findings deposed for a mesenchymal neoplasm.

The patient has performed an abdominal Computed Tomography contrast medium that shows a solid lesion with axial diameter of about 6 cm, with inhomogeneous densitometry, assuming contrast medium, surrounded by a thicker rim, containing gas bubbles, present in the pelvic, in continuity with the uterus, extending to the cranio-caudal about 9 cm.

The serum concentrations of cancer markers CEA, AFP, CA 19-9, CA 125, HE4, CA 15-3 are within the norm.

Cystoscopic examination revealed an urethra displaced upwards, bladder trigone raised significantly compared to standard as ab-extrinseco compression, but covered with mucosa of normal appearance.

Total abdominal hysterectomy, bilateral salpingo-oophorectomy, systematic pelvic lymph node dissection, omental biopsy and appendectomy were performed. On gross examination, a mass of about 8 cm deformed uterine profile. Ovaries and other pelvic organs grossly appeared normal. Gastrointestinal tract, paracolic gutters, liver, spleen, kidneys, and the undersurface of the diaphragm were free of lesions.

Surgery treatment was associated with adjuvant whole-pelvis radiation (45 Gy) and adiuvant chemotherapy (cisplatin/ifosfamide). After three month of follow up, the patient was free of tumor.

**Figure 1:**
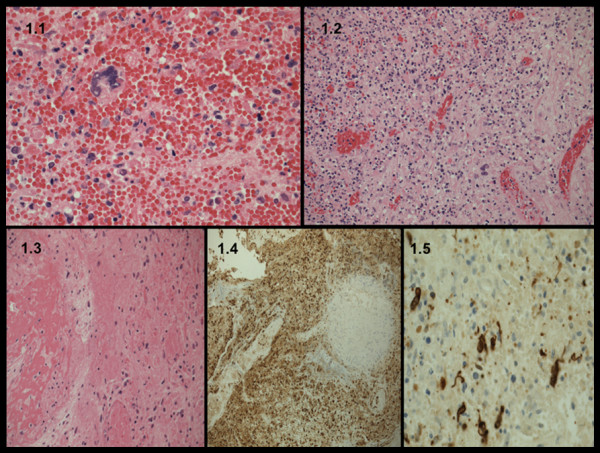
**Microscopic features and immunohistochemistry**. **1.1 **rhabdoid cells, **1.2 **rhabdoid cells with necrosis, **1.3 **mesenchymal stomal cell differentiation, **1.4 **vimentin immunohistochemistry, **1.5 **desmin immunohistochemistry.

On gross examination, a friable and polypoid lesion arised from uterine cervix and involved the cervical-isthmic mucosa and the endometrium. A focal chronic ulceration of the head of the polyp was noted. The cut surface of the tumor was fleshy and gelatinous. There was focal myometrial invasion. On microscopic examination, the tumor was characterized by intimate admixture of epithelial component and sarcomatous stromal component. The epithelial component was minimal and mostly without epithelial atypia; while the sarcomatous component was clearly predominant (pure sarcoma: 70%), of high grade, with a high mitotic rate (10 MFs/10 HPFS). In addition, heterologous pure sarcomatous areas were noted comprising of rhabdomyosarcoma. There was no lymph node metastasis or lymphatic vascular space invasion, but a non-neoplastic focal thrombosis of small blood vessels in the head of the polyp was noted. Omentum and appendix were negative for tumor localization. The cytoplasm of the stromal cells showed a strong positive reaction for vimentin and desmin, but was negative for smooth muscle actin. Also, tumor cells were negative for β-HCG, estrogen and progesterone receptors. Only the focal areas of stromal tissue were positive for MYF 4 and also glands and rare isolated cells were positive for Keratin (MNF116). (Figure 1)

## Conclusions

Uterine adenosarcomas are relatively rare tumors, whose incidence appears increased in the last years. This is probably due both to a better understanding of different anatomo-pathological aspects of uterine sarcomas (especially with the development of the immunohistochemistry) and to a possible exposure to other different predisposing factors, pelvic irradiation and the use of tamoxifen for breast cancer[15].

Although the etiologic factors are still unknown, at least three possible risk factors are currently under discussion:

- Pelvic irradiation, exceptional cases have been reported in patients with a history of previous pelvic irradiation; [16]

- The hyperestrogenism, such as after prolonged unbalanced estrogen stimulation or long term oral contraceptive use; [17]

- Treatment with tamoxifen, a small series has been reported after treatment with tamoxifen for breast cancer[18].

However, these may be coincidental associations and there are no proven etiologic factors. Anyway, our patient had none of these risk factors.

Uterine endometrial adenosarcomas were typically found in post-menopausal women with the median age at presentation of 58 years[7]. Compared with them, cervical adenosarcomas tended to appear more often in younger women with the average age at presentation of 31 years[19]. The most common presenting symptom is abnormal vaginal bleeding (71%), spotting or menorrhagia or metrorrhagia, as in our patient. These tumors can present as a pelvic mass (37%), uterine polyp (22%), or an enlarged uterus (22%)[11]. Pain, foul smelling vaginal discharge, or symptoms of pelvic pressure have also been reported. In the largest published series of Mullerian Adenosarcomas of the cervix, the most common finding is tissue protruding from the external cervical os and the clinical impression is frequently of benign endocervical polyps. A history of recurrent polyps on clinical and pathologic examination is typical before MA is finally diagnosed[7,19-24]. Typically, the glands are cystic and the stroma concentrates around them forming periglandular cuffs. The hystologic picture is reminiscent of a phyllodes tumor of the breast[1]. Using the WHO definition, stromal mitotic activity of two or more per HPFs is required for a diagnosis of adenosarcoma. [2,3] but in practice the diagnosis is made with stromal mitotic activity less than this if the characteristic architecture with periglandular cuffing is present, because the number of mitoses may be variable from area to area[14]. Adenosarcomas generally are low-grade neoplasms capable of local recurrence after polypectomy or hysterectomy and less commonly distant metastasis. The two most important adverse prognostic factors are deep myometrial invasion, as a predictor of aggressive behavior, and sarcomatous overgrowth, as a predictor of poor prognosis[14,25]. Myometrial invasion is found in 15% of cases, but deep invasion in only 5%.[7] in 8-54% of uterine and 30% of ovarian adenosarcomas, the sarcomatous overgrouth, defined as the presence of pure sarcoma, usually of high-grade and without a glandular component, occupying at least 25% of the tumor, has been reported[1]. The presence of heterologous elements, especially rhabdomyosarcoma, may represent a more clinically aggressive tumor. Most cases of MASO originate from the uterine corpus; while in the English literature only three cases of the MASO of uterine cervix have been described[26-28]. MASO of uterine corpus has a highly aggressive malignant potential; but aggressiveness of cervical MASO is uncertain because they are extremely uncommon, since its cervical location and the presence of heterologous elements are extremely infrequent. Others unfavorable prognostic factors, such as necrosis and extrauterine spread, have been recognized in the MA of the uterus[26]. The main differences between our case and other MASO cases reported in English literature are the presence of focal myometrial invasion in our case, and also the presence of heterologous elements in our case as in Duggal et al's case[28].

About the immunophenotype, smooth muscle actin and desmin may also be positive. In areas of high-grade sarcoma and of sarcomatous overgrowth, the mesenchymal component exhibits a higher MIB1 proliferation index and may be p53 positive. The immunophenotype is similar to that of an undifferentiated uterine sarcoma with usually loss of expression of the cell differentiation markers estrogen receptor, progesterone receptor and CD10[29,30]. Area of rhabdomyosarcoma express desmin and skeletal muscle markers, myogenin and myoD1. In adenosarcoma with sarcomatous overgrowth, the mesenchymal component may be DNA aneuploid whereas in adenosarcomas without sarcomatous overgrowth, it is usually DNA diploid[31].

The differential diagnosis of MASO of the uterine cervix should be made with caution. It includes benign lesions (such as adenofibroma, endocervical polyp, adenomyoma of the cervix) and malignant lesions (such as malignant mixed mullerian tumors, embryonal rhabdomyosarcoma). MA can be easily distinguished from adenofibroma (both epithelial and stromal components benign) using the criteria defined as unique to adenosarcoma such as, a marked degree of atypia of mesenchymal cells, a histological malignant element, the presence of myometrial invasion, and two or more mitotic figure per 10 HPF[7,25]. However, these features are not always present; thus, they are less applicable in the discrimination of malignant degree. Some cases presented with recurrent cervical polypoid lesions that were initially considered benign form, probably due to the irregular appearance of the stromal component, with normal areas adjacent to others with higher stromal density, mitotic activity, and atypia. In those cases, most patients subsequently return for recurrent endometrial and endocervical polyps, which are reinterpreted as adenosarcoma[7,19-23]. Adenomyomas can be distinguished from adenosarcoma by the presence of well-defined myomatous stroma. Malignant mixed mullerian tumors (MMTs) are also biphasic lesions but both the stroma and the epithelium are malignant. In accord to the uterine MASO, MMTs behave aggressively and often present with early recurrences and metastases[32,33]. Embryonal rhabdomyosarcoma (sarcoma botryoides), the most common malignant tumor of the vagina in infants and children, can occasionally present in the cervix[34,35]. It consists of a polypoid growth with densely cellular submucosal cambium layer and scattered rhabdomyoblasts. The stroma is edematous, not fibrous and the leaf-like pattern is absent. An important differentiating factor is the age at presentation, since embryonal rhabdomyosarcoma occurs at an earlier age and MA of the cervix occurs in an older age group.

The relative rarity of cervical MASO made the assessment of the most effective means of management difficult[9,19-23]. Most authors recommend total abdominal hysterectomy, usually accompanied by bilateral salpingo-oophorectomy[7,33]. Among gynecologists there is no common consensus on the value of staging by lymphadenectomy during primary surgery. In general, women with superficial MA or MA confined to a cervical focal area probably do not require radiation therapy, but those with tumors invading more than halfway through myometrium or with two or more unfavorable factors have a high likelihood of recurrence and might benefit from high-dose pelvic radiation with or without aggressive chemotherapy[20,23]. Local excision has been curative in rare cases, and could be preferred especially in young patients[7,23]. In their MASO case Park et al. [26] performed total abdominal hysterectomy and bilateral salpingo-oophorectomy with pelvic lymph node dissection. The patient didn't receive adjuvant therapies and underwent a regular follow-up. The patient was clinically free of disease after 9 months of surgery. Comunoğlu et al didn't perform lymph node dissection after total abdominal hysterectomy and bilateral salpingo-oophorectomy and the patient was free of disease for 14 months of surgery[27]. Duggal et al. performed total abdominal hysterectomy with bilateral salpingo-oophorectomy and omentectomy because of clinical and histopathological features of the mass suggestive of a possible cervical sarcoma. In addition, peritoneal washings were sent for cytological examination. Surgery treatment was followed by six cycles of chemotherapy and subsequent radiotherapy. After one year of follow up, there was recurrence of disease and the patient died[28]. About the management, the main difference between our case and the previous MASO cases reported in English literature is the surgical treatment including pelvic lymph node dissection and adjuvant chemo-radio therapy. Among gynecologists there is no consense on the practical value of staging by lymphadenectomy during primary surgery. In uterine neoplasm with a sarcomatous component the most important prognostic factor generally is the stage[11,20,36-38]. The lymphatic vascular space invasion seems a relevant prognostic factor with an impact on overall survival and distant metastasis-free survival but only in the early stage of the disease [9,39]

In conclusion, we present an extremely rare case of cervical MASO, our knowledge the fourth case reported in the English literature for its location and the second MASO case of the cervix with heterologous elements. (Table 1)

**Table 1:** Summary of MASO cases reported in English literature

Case	Age	Myometrial invasion	Heterologous element	Immunophenotype	Lymphnode metastasis/ vascular invasion	Surgical treatment	Adjuvant treatment
**Park H.M. et al case **[3]	37	No	No	**Vimentin**: ++ **CD34: **- **HMB-45: **- **Desmin: **- **Cytokeratin: **- **S-100: **- **CD99: **- **SMA: **+/-	No	TAH + BSO+ PLA	No

**Comunoğlu N. et al case** [4]	60	No	No	**Vimentin: **++ **CD34: **- **HMB-45: **- **Cytokeratin: **- **S-100: **- **Pg: **+ **E: **+ **SMA and Desmin:** + in sarcomatous stromal cell	No	TAH + BSO	No

**Duggal R. et al case **[5]	15	No	Yes: chondrosarcoma, myxoid liposarcoma, leiomyosarcoma, rhabdomyosarcoma	**Vimentin: **+ in low grade sarcomatous areas; ++ in high grade sarcomatous areas **CD10: **+ in low grade sarcomatous areas **S-100: **+ in liposarcomatous areas **Pg: **- **E: **-	-	TAH + BSO + O + PW	CHT RT

**Present case**	72	Yes: focal invasion	Yes: rhabdomyosarcoma	**Vimentin: **++ **keratin (MNF 16): **+/- **MYF4: **+/- **B-hCG**: - **Pg: **- **E: **- **SMA: **-	No	TAH + BSO+ PLA O + PW + A	CHT RT

**+: **positive

**++: **strongly positive

**-: **negative

**+/-: **positive in focal areas

**SMA: **smooth muscular actin

**Pg: **progestogen

**E: **estrogen

**TAH: **total abdominal hysterectomy

**BSO: **bilateral salpingo-oophorectomy

**PLA: **pelvic lymphadenectomy

**O: **omentectomy

**PW: **peritoneal washing

**A: **appendectomy

**CHT: **chemotherapy

**RT: **radiotherapy

In uterus, adenosarcoma with sarcomatous overgrowth are aggressive tumors frequently associated with postoperative recurrence or metastases, and poor prognosis, markedly contrasting with typical mullerian adenosarcoma of the uterus. Myometrial invasion and heterologous elements seem to be principle prognostic factors[26]. Aggressive behavior of MASO of the uterine cervix is uncertain because they are extremely uncommon. The rarity of the cases of cervical MASO and the variety of the presenting symptoms (such as abnormal vaginal bleeding, pelvic mass, abdominal pain, recurrent cervical polyps), make difficult the differential diagnosis and so the optimal therapy is uncertain. More case reports and perspective studies are needed for determining the treatment options for cervical MASO with heterologous elements.

## Consent

Written informed consent was obtained from the patient for publication of this case report. A copy of the written consent is available for review by the Editor-in-Chief of this journal.

## Competing interests

The authors declare that they have no competing interests.

## Authors' contributions

TSP and SG made substantial contribution to conception and design. SDG and GG have been involved in drafting the manuscript. MR revised it critically for important intellectual content. GBN gave final approval of the published version. All authors read and approved the final manuscript.

## Pre-publication history

The pre-publication history for this paper can be accessed here:

http://www.biomedcentral.com/1471-2407/11/236/prepub
